# Salted Meats

**Published:** 1883-04

**Authors:** 


### SALTED MEATS.

We do not object to salted meats as an item in any bill of fare; they should indeed, occupy an important place in the list of foods, but when, as often happens, particularly in remote and sparsely settled districts, all the animal food to be obtained is salted, it becomes mainly responsible for the great prevalence of scorbutic diseases in such districts. The evil effects that are liable to follow a long continued use of salted meats may be modified to a great extent by the free use, at the same time, of fresh vegetables and acid fruits.

The effect of an over use of such foods—as, for example, salted pork—seems to be to introduce an excess of salt into the system, thus changing the natural condition of the blood by an excess of one of its elements, and producing obstructions in the eliminating process; unsightly sores often appear, particularly on the face, which is kept dry and cool by exposure to the air, thus closing the pores of the skin by contraction of their orifices; the result being that the fluid, unable to pass out through the skin, becomes blocked up in the pores, causing inflammation at certain points; then ulceration follows in the form of pimples. These discharge, heal up and, if nothing is done to prevent it, are succeeded by other crops at various intervals.

Salted foods are not so digestible as fresh foods, as a rule; but that is a difficulty easily remedied, under the rule of moderation. The stomach, like all other organs of the body, will adapt itself to all reasonable demands, and will, in fact, tolerate a good deal of harsh treatment for a long time; but sooner or later resents abuses.

The best plan is to live on a mixed diet of vegetable and animal foods, with frequent changes of variety. It is well to have a special bill of fare for each day in the week, to include fruits and other foods that are in season.

